# Structural and Phylogenetic In Silico Characterization of *Vitis vinifera* PRR Protein as Potential Target for *Plasmopara viticola* Infection

**DOI:** 10.3390/ijms25179553

**Published:** 2024-09-03

**Authors:** Sofía M. Martínez-Navarro, Xavier de Iceta Soler, Mónica Martínez-Martínez, Manuel Olazábal-Morán, Paloma Santos-Moriano, Sara Gómez

**Affiliations:** Innovative Seed Lab (ISL), Faculty of Biomedical and Health Sciences, Universidad Europea de Madrid, 28670 Villaviciosa de Odón, Spain; sofia.mrtnez@gmail.com (S.M.M.-N.); xavierdeiceta@gmail.com (X.d.I.S.); monica.martinez@universidadeuropea.es (M.M.-M.); manuel.olazabal@universidadeuropea.es (M.O.-M.); sara.gomez@universidadeuropea.es (S.G.)

**Keywords:** lectin domain, Pattern Recognition Receptors (PRRs), lectin receptor-like kinase (LecRLK), *Vitis vinifera*, *Plasmopara viticola*, downy mildew, carbohydrate recognition

## Abstract

Fungi infection, especially derived from *Plasmopara viticola*, causes severe grapevine economic losses worldwide. Despite the availability of chemical treatments, looking for eco-friendly ways to control *Vitis vinifera* infection is gaining much more attention. When a plant is infected, multiple disease-control molecular mechanisms are activated. PRRs (Pattern Recognition Receptors) and particularly RLKs (receptor-like kinases) take part in the first barrier of the immune system, and, as a consequence, the kinase signaling cascade is activated, resulting in an immune response. In this context, discovering new lectin-RLK (LecRLK) membrane-bounded proteins has emerged as a promising strategy. The genome-wide localization of potential LecRLKs involved in disease defense was reported in two grapevine varieties of great economic impact: Chardonnay and Pinot Noir. A total of 23 potential amino acid sequences were identified, exhibiting high-sequence homology and evolution related to tandem events. Based on the domain architecture, a carbohydrate specificity ligand assay was conducted with docking, revealing two sequences as candidates for specific *Vitis vinifera–Plasmopara viticola* host–pathogen interaction. This study confers a starting point for designing new effective antifungal treatments directed at LecRLK targets in *Vitis vinifera*.

## 1. Introduction

Understanding the interaction between plants, pathogens, and the underlying immune mechanisms they can trigger is of vital importance to address current societal issues related to food security and sustainability. This comprehension is essential for developing environmentally friendly and more effective disease management strategies [1]. Among the plants affected by these issues, the grapevine stands out for its versatility in the industry and its economic importance.

Grapes are the third most important crop in the world of agriculture. They are primarily cultivated in areas with a Mediterranean climate, with China, Italy, the United States, France, and Spain being the main producers [2]. Grape production is almost entirely focused on wine grapes, with *Vitis vinifera* being one of the most cultivated species. Within this species, there are different varieties valued for their unique contributions to the final product. Among the most widespread varieties of *Vitis vinifera* are Chardonnay and Pinot Noir due to the organoleptic characteristics they confer on the wine [3,4]. 

As mentioned earlier, grapes are one of the most widely cultivated and commercially important fruits. However, they are also one of the most susceptible crops to weather changes and various diseases. Among these diseases, grapevine downy mildew, caused by the oomycete *Plasmopara viticola*, is of great significance. Downy mildew is classified as one of the main causes of annual crop losses, especially in European fields, where most plantations belong to the *Vitis vinifera* family, which is susceptible to this pathogen [5]. *Plasmopara viticola* typically develops in areas with low rainfall during spring and summer, especially if temperatures are below average in spring [6]. Downy mildew affects the green tissues of the plant, mainly the leaves, thereby reducing their nutritional capacity and their gas exchange surface, resulting in a decrease in production rate and grape quality [5,7]. 

When a pathogen breaches the physical barrier, the first layer of defense is constituted by extracellular receptors, known as PRRs (Pattern Recognition Receptors). These extracellular receptors detect molecules that are conserved in most pathogens, which are called PAMPs (Pathogen-Associated Molecular Patterns) or MAMPs (Microbe-Associated Molecular Patterns) [1]. Additionally, PRRs detect Damage-Associated Molecular Patterns (DAMPs), allowing the plant to react to physical damage caused by the pathogen [8]. 

Within PRRs, two groups can be observed: RLPs (Receptor-Like Proteins) and RLKs (receptor-like kinases), depending on whether they have a kinase domain. Belonging to the RLK family, well-characterized lectin-RLK (LecRLK) lectin-like proteins with carbohydrate-binding functions are widely distributed in plants [9]. LecRLKs can be further classified into three types: type L, type G, and type C [10]. Type L lectins contain an extracellular legume lectin-like domain, which contains a hydrophobic cavity for binding hydrophobic ligands. This carbohydrate-binding function positions these proteins as potential primary candidates for recognizing oomycetes, as these often present such molecules on their surface [11]. 

After the detection of PAMPs by PRRs, their kinase domain triggers the signal transduction cascade, resulting in the upregulation of certain genes involved in PTI (Pattern Triggered Immunity) [9,12]. Subsequently, the signal produced by the PTI response is amplified through the synthesis of Salicylic Acid (SA), triggering a similar systemic response known as Systemic Acquired Resistance (SAR) [13]. However, sometimes, this response is not strong enough to fight an infection. For this reason, in agricultural plantations, pesticides and other measures are often employed to treat these diseases.

Currently, chemical treatments are the only way to control *Plasmopara viticola*, with copper-based fungicides being the most used and effective against the disease. However, high amounts of copper can affect the microbiota present in the cultivation field, altering its composition and causing imbalances in the soil nutrients [14]. This alteration can lead to a loss of fertility, resulting in a significant loss of cultivation area and, consequently, substantial economic losses. Moreover, most of these fungicides do not penetrate the green tissues but act on the surface, while the pathogen develops in deeper layers of these tissues [6]. This inefficacy, along with the environmental and human health hazards of these fungicides, highlights the need to seek new treatments for the disease.

Nowadays, crossbreeding resistant varieties with those of interest is the main non-pesticide technique for improving plant immune responses to pathogens. However, as mentioned earlier, the different organoleptic characteristics conferred by specific grape varieties affect the quality, perception, and value of the final product. Thus, as crossbreeding could alter the characteristic aromas and flavors of the cultivated grape variety, they are generally avoided as a technique in the viticulture industry, where conserving the different phenotypes and genotypes is especially important [3]. 

Therefore, studying pathogenesis and pathogen recognition is of vital importance to develop new targeted treatments that do not require major genetic modifications to avoid altering the organoleptic characteristics of the final product [2]. 

For this reason, the objective of this work is to conduct a structural bioinformatics study of PRR proteins in *Vitis vinifera* non-resistant varieties and their interaction with the pathogen *Plasmopara viticola* to expand the current knowledge about them for the subsequent study and development of techniques to combat the infection in a selective and specific manner.

Despite the information already available on grapevine–pathogen interactions, this study introduces a novel approach by focusing on the structural bioinformatics of PRR proteins in *Vitis vinifera*. Previous studies have largely focused on broader aspects of plant immune responses or specific interactions at the molecular level. However, the aim of this work is to delve deeper into the structural and functional aspects of LecRLKs in *Vitis vinifera*, particularly their interaction with *Plasmopara viticola*. This approach not only expands the current knowledge but also lays the groundwork for developing more selective and effective antifungal treatments. 

## 2. Results

### 2.1. Vitis vinifera LecRLK Identification and Classification

Based on the gene ontology (GO) approach, 61 initial amino acid sequences of proteins encoded by the genome of *Vitis vinifera* and related to oomycete defense were initially identified. 

Duplicated sequences were manually removed, and to predict the presence/absence of lectin and kinase domains, the remaining full-length amino acid sequences were submitted to Pfam and ScanProsite for analysis [15,16]. Sequences associated with oomycete defense that lacked any structural domains were observed. Therefore, these sequences were no longer included in the study, resulting in a total of 43 sequences, which were analyzed in detail. With the information collected in UniProt, they were assigned according to the plant variety, which could be Pinot Noir or Chardonnay, as both varieties are susceptible to *Plasmopara viticola* infection. The sequences that could not be assigned to any particular variety were eliminated from the study, and finally, 23 out of 43 remained, which are the ones presented in this work. Given that the 23 selected sequences were lectin-like proteins, an exploration was conducted to determine whether they could be of a G, C, or L type. For this purpose, an alignment matrix was performed, determining the percentage of similarity of each sequence and using as models for each type the corresponding coding sequence in *Arabidopsis* (AT1G65790 as the G type with the Q39086 UniProt code; AT1G52310 as the C type with the Q9C823 UniProt code; and AT2G37710 as the L type with the O80939 UniProt code) from the SwissProt database. By analyzing the alignment matrix, all proteins were assigned to the L-lectin family (see Tables S1 and S2). These results are conclusive with the findings obtained in Pfam, where the amino-terminal region of all the sequences in the study was assigned to the PF00139 family, which validates the L-lectin-type classification. 

### 2.2. Chromosomal Characterization

The next step was to carry out chromosomal characterization by mapping the protein-coding genes. For that purpose, full-length amino acid sequences were used to perform a BLASTX search. For comparison, two different databases were employed: Ensemble Plants, with the *Vitis vinifera* genome version PN40024.v4, and Phytozome, using the *Vitis vinifera* genome version v2.1. In both cases, the entries with the lowest E-values and the highest percentage of sequence similarity were selected, covering most of the sequence. A comprehensive table for each variety provides detailed information for each LecRLK gene, including its UniProt ID, DNA strand, chromosome localization, start and end nucleotide, CDS (bp), ORF (aa), transcript ID from the Ensembl database (PN40024.v4), and gene identifier from the Phytozome database (*Vitis vinifera* genome ID v2.1) (see Table 1 and Table 2). As can be seen, in two Chardonnay genes and one Pinot Noir gene, no corresponding transcript IDs were found in the Phytozome database. 

**Figure 1:**
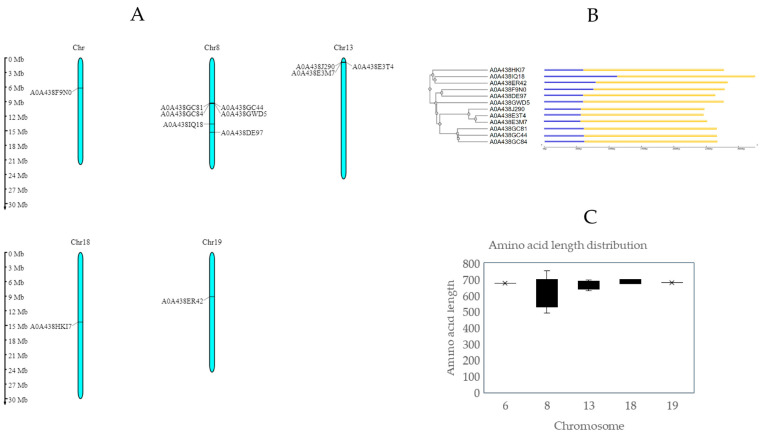
Chromosomal distribution of LecRLK genes in the genome of *Vitis vinifera* Chardonnay variety. (**A**) Chromosomal location of proposed LecRLK genes in the *Vitis vinifera* genome obtained with MG2C [23]. (**B**) Phylogenetic tree obtained with ClustalW, and exon–intron distribution of LecRLK genes performed with gene structure display server. Legend: Yellow boxes represent CDS sequence, blue boxes represent UTR sequence, and black lines represent introns [24,25]. (**C**) Amino acid length distribution of LecRLKs in Chardonnay variety.

**Figure 2:**
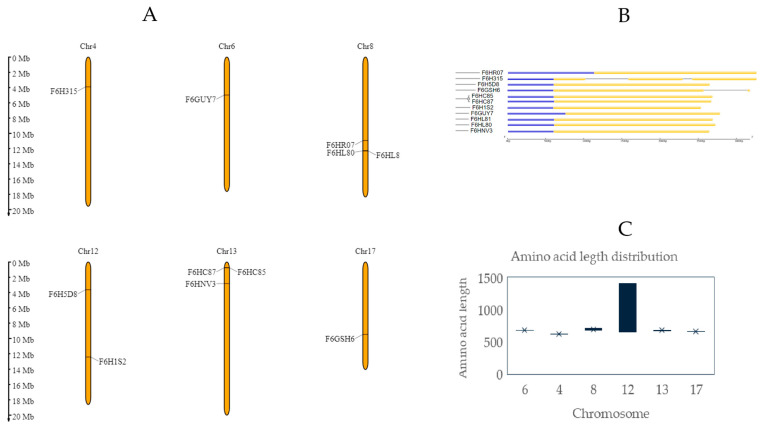
Chromosomal distribution of LecRLK genes in the genome of *Vitis vinifera* Pinot Noir variety. (**A**) Chromosomal location of proposed LecRLK genes in the *Vitis vinifera* genome obtained with MG2C [23]. (**B**) Phylogenetic tree obtained with ClustalW, and exon–intron distribution of LecRLK genes performed with gene structure display server. Legend: Yellow boxes represent CDS sequence, blue boxes represent UTR sequence, and black lines represent introns [24,25]. (**C**) Amino acid length distribution of LecRLKs in Pinot Noir variety.

Genes were present in 8 of the 19 chromosomes that constitute the *Vitis vinifera* genome. In Chardonnay and Pinot Noir, some chromosomal locations on chromosomes 8 and 13 contain multiple genes clustered together. This tandem disposition is also reported in *Populus*, *Brassicaceae*, and other genomics research previously undertaken on *Vitis vinifera* and represents the main mechanism of lectin family expansion [17,18,19]. Together with this phenomenon, segmental duplication was considered one of the main reasons that could explain the high density of LecRKL genes in plants, as described in rice, soya, or *Arabidopsis* [20,21,22]. Furthermore, phylogenetic analysis reveals that in the Chardonnay variety, three out of six strongly conserved sequences were located in chromosome 8 forming a cluster. In contrast, sequence A0438GWD5, also located in chromosome 8, was mapped in a different region. Another phylogenetic cluster was observed, with three out of three tandem repetitions mapped in chromosome 13 (Figure 1A). In the case of the Pinot Noir variety, just two out of three genes belonging to chromosome 13 were phylogenetically grouped (Figure 2A). 

An exon–intron analysis reveals considerable conservation in the intron number and gene size (Figure 1B, Figure 2B, Figure 1C, and Figure 2C, respectively). Figure 1B and Figure 2B represent, in a schematic way, the exon–intron distributions, as well as the UTR sequences in all coding sequences. No introns were observed in the Chardonnay variety and ranged from one to two in Pinot Noir. A low level of intron structures was previously observed in other L-type genes, reinforcing the genetic ancestor relationship between all LecRLK genes identified [19].

Through the use of the PlantCARE database, analyses of the cis-acting elements in A0A438E3M7, A0A438HKI7, A0A438J290, F6HC85, F6HC87, and F6HVN3 were undertaken (see Table S3). In this study, 23 putative cis-elements showing a 10-value maximum matrix score were identified and further characterized into four groups: hormonal regulation, stress regulation, light regulation, and plant development. Among these, light and hormonal responses are the most prevalent (34 and 16 occurrences, respectively), especially G-box (light-related) with 12 occurrences. Despite showing a lower global occurrence (with just seven), ARE (a cis-element related to stress regulation) has a six-value occurrence. 

### 2.3. Protein Domain Architecture 

**Figure 3:**
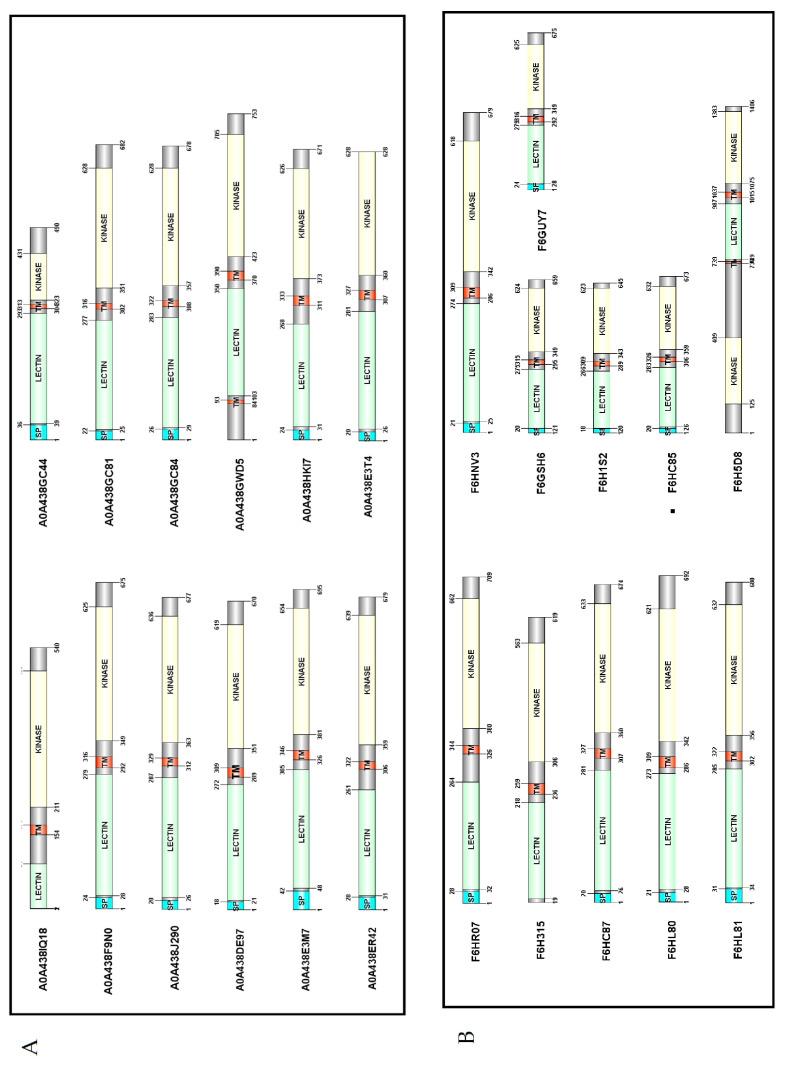
Domain architecture of LecRLKs from *Vitis vinifera*. (**A**) Chardonnay proteins and (**B**) Pinot Noir proteins. SP (light cyan color): signal peptide; TM (red color): transmembrane domain; Lectin (green color): legume lectin domain (Pfam 00139); and kinase (light yellow color): kinase domain (Pfam IPR011009). Created with DOG 2.0 software [26].

Full-length amino acid sequences were submitted to Pfam, Interprosite, THMM, and signalIP websites to analyze the domain architecture. Based on the results obtained, Figure 3 was elaborated with DOG 2.0 software [26]. In general terms, most of the predicted sequences had a similar amino acid length, with the exception of F6H5D8. As can be observed, 10 out of 12 sequences from Chardonnay present signal peptide regions (83% of total), and 1 sequence presents 2 transmembrane domains. The majority of the detected sequences follow typical L-lectin architecture, with an extracellular lectin domain, a transmembrane domain, and an intracellular kinase domain. According to the classification proposed by Yang et al., typical LecRLKs can be classified as Class I on the basis of the number and orientation of their transmembrane domains [17]. Interestingly, A0A438GDW5 comprises two transmembrane domains and can be rated as Class VI (Figure 3A). However, F6H5D8 shows two intracellular kinase domains, two transmembrane domains, and just one lectin domain (Figure 3B), which means that no correspondence with any previously defined class based on the transmembrane domain organization was detected. 

**Figure 4:**
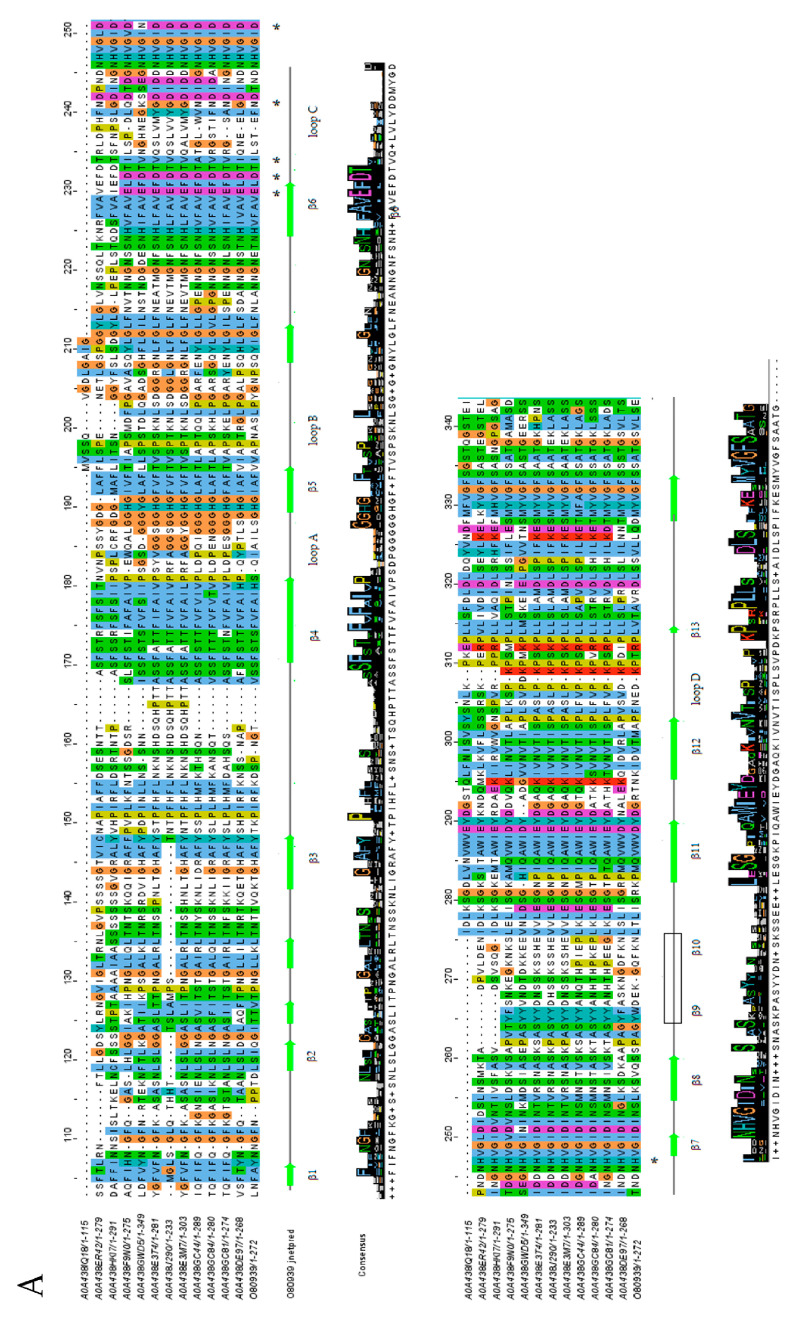

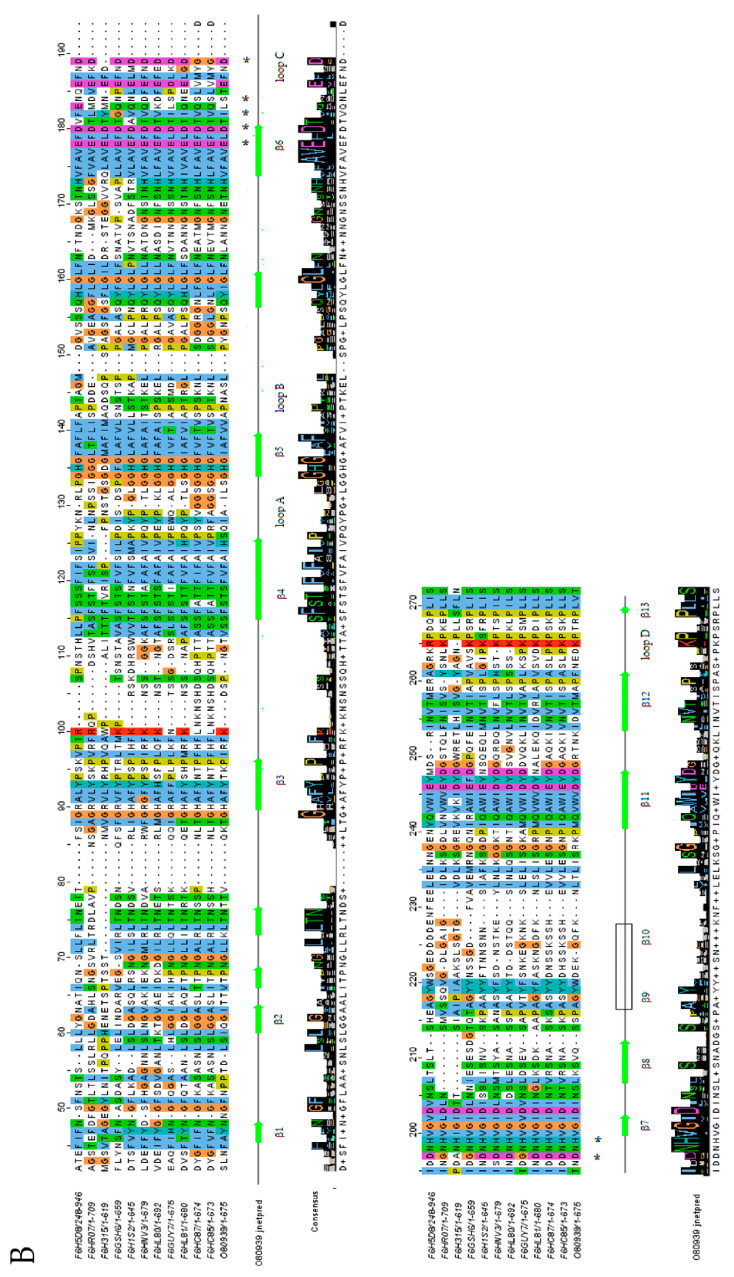
Multiple sequence alignment of LecRLK legume lectin domain sequences obtained with ClustalW and colored as Clustal codes. Predicted secondary structure of O80939 UniProt code protein was obtained with Jprep for comparison, and consensus logo sequence is shown at the bottom. β-strands (numbered β1–β13) are displayed as green arrows and the α-helix as red regions. Loops A–D are included in secondary structure. Essential amino acids involved in carbohydrate recognition are highlighted with an asterisk. (**A**) Chardonnay variety; (**B**) Pinot Noir variety [25,29,30].

The lectin domain followed the corresponding concanavalin A-like lectin domain superfamily folds, with 13 antiparallel β-strands in 2 sheets, constituting a widely conserved sandwich structure (Figure 4A,B) [27,28]. 

The four loops that interconnect β-strands have been described as critical for carbohydrate recognition and cation coordination, essential for the stability of oligosaccharide binding sites [31]. In particular, some amino acids present in loop C have been described as critical for carbohydrate stabilization in the recognition site and can be observed as commonly conserved. Surprisingly, A0A438IQ18 reveals an incomplete lectin domain, with just the last β-strands present. In all cases, β9–β10 were not correctly predicted in the secondary structure, but subsequent studies have confirmed their presence. 

**Figure 5:**
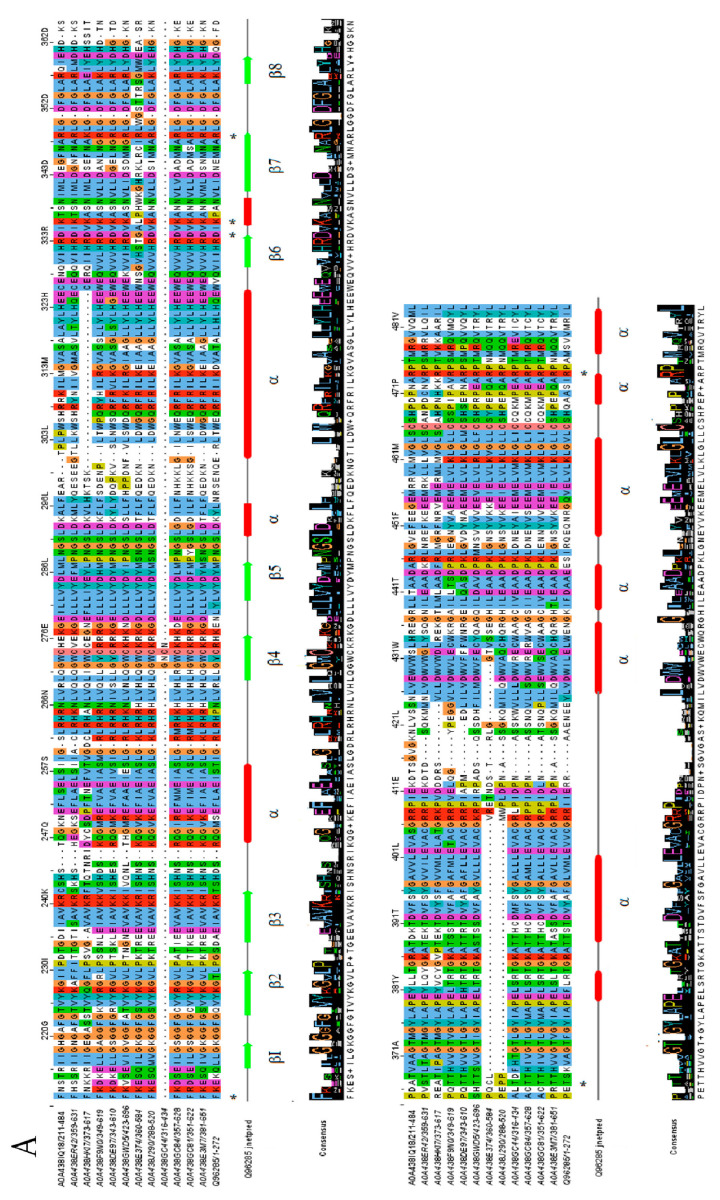

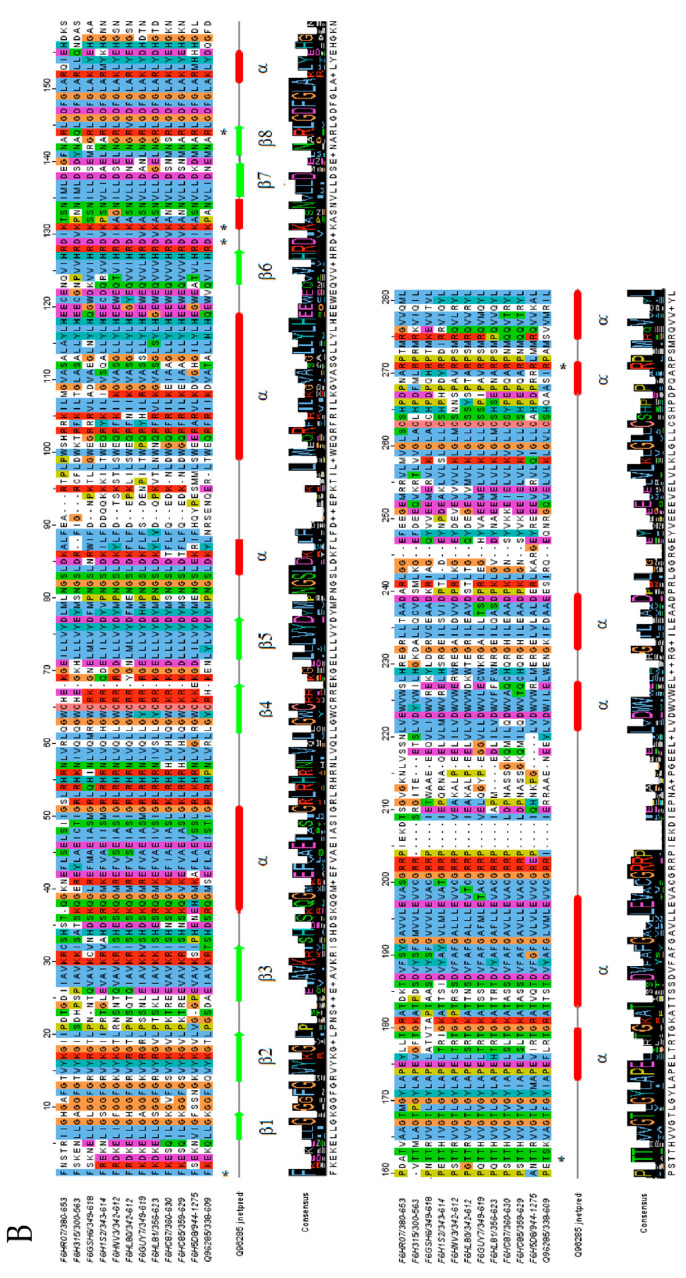
Multiple sequence alignment of LecRLK domain sequences obtained with ClustalW and colored as Clustal codes. Predicted secondary structure of Q96285 UniProt code protein was obtained with Jprep for comparison, and consensus logo sequence is shown at the bottom. β-strands are displayed as green arrows and the α-helix as red regions. Essential amino acids involved in catalytic activity are highlighted with an asterisk. (**A**) Chardonnay variety; (**B**) Pinot Noir variety [25,29,30].

It is well known that the kinase domain is essential for the signal transduction of LecRLK-downstream-mediated pathways [32]. Some residues have been identified as critical for enzyme activity, but some discrepancy still exists concerning their classification to a specific kinase family. Ser/Thr kinase proteins contain a conserved sequence of DIKPAN instead of DLARRN between β6 and β7 (known as the subdomain VIb) [28,33]. As could be observed, despite the Chardonnay and Pinot Noir sequences containing critical Asp and Lys residues conserved in most of the cases (except the A0A438ET4 and A0A438GC44 sequences), no defined kinase family could be correctly assigned (Figure 5A,B). In this respect, some authors have proposed the possibility that some LecRLK proteins could lack kinase activity or even present dual specificity, but further studies are needed to corroborate this possibility [34,35].

### 2.4. Carbohydrate Specificity Analysis through Docking

In order to explore the carbohydrate recognition pattern, 3D molecular structures of proteins were obtained with a Phyre2 modeling-based homology server, and based on lectin prediction, proteins were classified according to their carbohydrate candidate ligand specificity (Table 3) [36]. OMIC analysis reveals that a huge amount of oomycetes’ own membrane carbohydrate structures were derived from GalNAc [34]. For this reason, as a proof of concept, docking analysis with GalNAc specificity models was performed. 

All sequences showing exclusively potential GalNAc affinity (A0A438E3M7, A0A438HKI7, and A0A438J290, F6HC85, F6HC87, and F6HNV3) were selected, and lectin domains were resubmitted for modeling with Phyre2, and PDB files obtained were used for docking analysis. Despite the predicted F6H5D8 GalNAc specificity, it was excluded from the analysis due to its atypical structure and dual model result.

Initially, a 3D structure of N-acetyl-galactosamine (GalNAc) with the minimization of energy was generated with CORINA Classic (https://demos.mn-am.com/corina_interactive.html (accessed on 1 July 2024)) and Antechamber software v22.2, and using Autodock/Vina’s default settings, the potential binding sites for GalNAc within the already generated models were searched for [44,45,46]. 

After a thorough examination of each of the possible conformations of GalNAc, only three out of the six starting models yielded comprehensive results. In the cases of A0A438E3M7, F6HC85, and F6HC87, at least one of the resulting molecules was close to the conserved residues (which are associated with ligand interactions) while also being in a similar position to its corresponding PDB counterpart (2D3S code). Of all possible conformations, the docked GalNAc with the aforementioned characteristics, as well as the highest affinity amongst them, was selected as the most likely for in vivo recognition (Table 4). 

**Figure 6:**
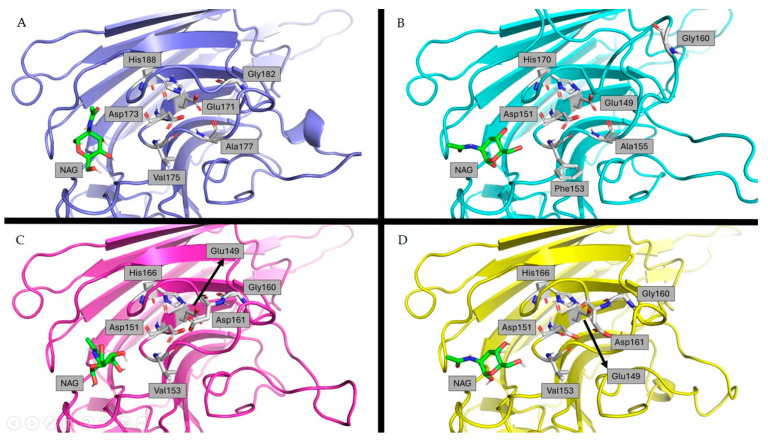
Cartoon representation of LecRLKs modeled with Phyre2 and represented by Pymol. Amino acids implicated in carbohydrate stability are shown as sticks, and GalNAc ligands are shown as green sticks. (**A**) A0A438E3M7; (**B**) A0A438J290; (**C**) F6CH85; (**D**) F6CH87 [47].

Through this analysis, a common pattern was discovered in the positions that GalNAc can adopt amongst these four models. A loop containing six strongly preserved amino acids within the different sequences (Figure 6) in close proximity to GalNAc is a commonality, although it may vary in its position from structure to structure. 

**Figure 7:**
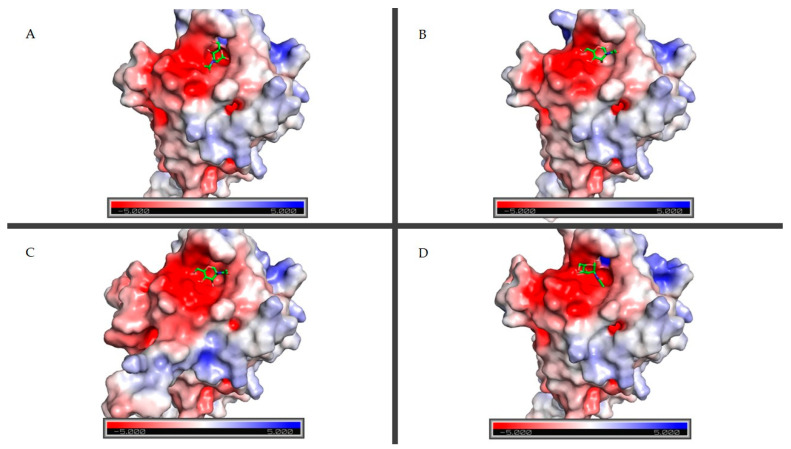
Surface electrostatic potential calculated by PyMOL. A positive charge is shown in blue, and a negative charge is shown in red. (**A**) A0A438E3M7; (**B**) A0A438J290; (**C**) F6CH85; (**D**) F6CH87. GalNAc ligands are represented by green sticks [47].

Moreover, when looking at the molecular electrostatic potential map (Figure 7), there seems to be a binding site that maintains a similar surface between the different modeled structures. In the case of A0A438E3M7 and F6CH87, they also have regions with both strong positive and negative charges, while F6CH85 only shows the latter. 

Although this does not confirm the molecular mechanism for the said interaction, it is relevant when it comes to determining it through further research.

## 3. Discussion

Grapevine cultivars, intrinsically associated with human activity, are severely affected by the oomycete *Plasmopora viticola*, causing significant economic losses worldwide. Multiple efforts are being made to understand how host–pathogen recognition mediates, and from multiple protein receptor candidates, LRK proteins are strongly considered key [6,48,49,50]. 

Legume lectins are closely related proteins that share high sequence similarity and highlight a pattern of evolutionary conservation with the aim of conserving their critical biological functions [21,32]. Their specific interaction with carbohydrates and their role in disease defense has attracted the attention of multiple research studies [9]. 

This study, which focused on the determination of candidate lectin proteins involved in oomycete recognition, has led to the discovery of 23 coded sequences within the *Vitis vinifera* genome. These genes were further categorized into Chardonnay or Pinot Noir grapevine varieties, and the present research encompasses the aspect of chromosome classification, evolutionary relationships, gene structure, and protein architecture. Finally, to open possible avenues to future research regarding the role of lectin-type receptors in the recognition of *Plasmopara viticola*, a docking experiment was conducted. 

Based on the phylogenetic results, LecRLKs showed a high conservation rate. Intron-less tendency and tandem-repeated sequences were observed, corroborating the fact that lectin protein expansion, as well as other genes involved in defense and disease resistance, is mainly due to tandem duplication events, possibly mediated as a response of plant-specific adaptation to biotic/abiotic stress [51,52]. 

Based on our results of the prediction of the domain architecture, most sequences were assigned to Class I (an extracellular legume–lectin domain, one transmembrane membrane interconnecting, and an intracellular kinase domain). However, one potential Class VI protein and another without a defined classification were also identified. Multiple sequence alignments allowed the definition of a typical LecRLK structure with conserved lectin and kinase domain structural conformation [28]. A clear kinase family assignation was not obtained, opening the possibility of being related to carbohydrate recognition but no further downstream signal transduction. The functional role of these results remains undefined but is in the same direction that was previously observed in other *Vitis* sp. research [17]. Cis-acting regulatory elements are known to be associated with the regulation of critical plant functions, and the usage of specific regulatory elements can be related to gene function [53]. Our results indicate that LecRLKs may play a key role in responding to environmental stimuli, as was previously suggested by other authors in other plant species [54,55].

In host–pathogen recognition, and particularly in *Plasmopara vinicola* infection, carbohydrate-specific interaction on the fungi cell wall and LecRLK plant proteins could inhibit oomycete growth and even initiate the signaling pathways of plant response [56]. For this reason, the identification of potential membrane-bounded proteins with key roles in this specific recognition is crucial. As a proof of concept, our docking analysis proposed three potential LecRLK candidates with affinity values under considerable levels using GalNAC as a ligand. To our knowledge, no structural analysis has been previously conducted with LecRLKs from *Vitis vinifera* and *Plasmopara vinicola* cell wall carbohydrates. In silico studies of the potential interaction between *Arabidopsis thaliana* proteins and *Hyaloperonospora arabidopsidi* fungi carbohydrates revealed the potential application of docking studies in the understanding of fungi–plant recognition [57]. 

Overall, these results provide useful information for the exploration of specific LecRLK *Vitis vinifera* proteins as potential biomarkers for the development of new eco-friendly treatments in *Plasmopara viticola* infection. Designing host-specific LecRLK plant defense elicitors, defined as compounds or microorganisms that trigger plant immunity through a cascade of signaling, is a promising strategy widely reported in the bibliography and which can be explored to be further applied alone or in combination with biostimulants to enhance their potential in crop protection. For example, applying mimetic-specific membrane-bounded molecules, such as GalNAc, known to be present in pathogens, can help in disease resistance [58,59,60,61,62]. Nevertheless, achieving these applications will require a more thorough investigation into the underlying signaling processes and interactions.

## 4. Materials and Methods

### 4.1. LecRLK Sequence Homolog Search in Vitis vinifera

For sequence retrieval and identification, the gene ontology (GO) approach was used, and the ontologies related to the taxon (29760, corresponding to *Vitis vinifera*), gene product (protein), and GO terms (0002229, corresponding to defense to oomycetes) were determined [63,64]. All results were consulted with the Quick-GO database (https://www.ebi.ac.uk/QuickGO/api/index.html (accessed on 2 March 2024)) of the European Bioinformatics Institute (EMBL-EBI), and amino acid sequence results provided by the SwissProt and TrEMBL databases were considered [63,65]. 

To identify potential lectin-type proteins, AT1G65790 (a G-type Arabidopsis LecRLK, Q39086 UniProt code), AT1G52310 (a C-type Arabidopsis LecRLK, Q9C823 UniProt code), and AT2G37710 (an L-type Arabidopsis LecRLK, O80939 UniProt code), were used as queries. A BLAST matrix initial alignment was obtained, and all sequences over 30% amino acid sequence similarity were assigned to each group. Sequences with two alignments over 30% amino acid sequence similarity were individually analyzed, and the one with the highest percentage was selected and considered as LecRLK candidate protein. 

### 4.2. Functional Domain Annotation and Functional Motif Prediction of LecRLKs

To predict protein functional motifs and domains, the full-length amino acid sequences of LecRLK proteins were subjected to Pfam and ScanProsite analysis with default settings. Proteins were further characterized based on the legume lectin domain (PF00139) and/or kinase domain (IPR011009). Duplicated sequences or those lacking a lectin domain or a kinase domain were not considered for further analysis [15,16]. 

To assess the number and location of transmembrane (TM) domains and signal peptides, full-length amino acid sequences were analyzed using TMHMM web-based software v2.0 (https://services.healthtech.dtu.dk/services/TMHMM-2.0/ (accessed on 31 March 2024)) and SignalIP v6.0 (https://services.healthtech.dtu.dk/services/SignalP-6.0/ (accessed on 1 April 2024)), respectively. When the TM motif was also predicted as a potential signal peptide, preference was given to signal peptide prediction. Domain maps were drawn using DOG 1.0 software [26,66,67]. 

Multiple sequence alignment was performed with ClustalW employing default settings, and secondary structure prediction was obtained with Jprep, with both integrated into Jalview software v2.11.3.3 [29,30,65]. In order to facilitate the analysis, lectin and kinase domains were separated and aligned with O80939 or Q96285 sequences, respectively. 

### 4.3. Chromosomal Location and Exon–Intron Distribution

Genes of the candidate proteins previously selected were mapped to grapevine chromosomes based on positions in the Grape Genome website (PN40024.v4) (https://plants.ensembl.org/Vitis_vinifera/Info/Index (accessed on 25 March 2024)) and Phytozome (https://phytozome-next.jgi.doe.gov/ (accessed on 25 March 2024)), and Chardonnay or Pinot Noir *Vitis vinifera* strains were assigned to each amino acid sequence. 

Exon–intron analysis was performed using the online Gene Structure Display server 2.0 (https://gsds.gao-lab.org/ (accessed on 21 May 2024)) [24].

To investigate the cis-acting regulatory elements in A0A438E3M7, A0A438HKI7, A0A438J290, F6HC85, F6HC87, and F6HVN3 sequences, a 2000 bp promoter region upstream from each gene was extracted employing the Phytozome tool. Following, the PlantCARE database (https://bioinformatics.psb.ugent.be/webtools/plantcare/html/ (accessed on 27 August 2024)) was then utilized to analyze the cis-regulatory elements, and the ubiquitous elements were filtered [68,69].

### 4.4. Multiple Sequence Alignment and Phylogenetic Analysis

Full-length amino acid sequences of LecRLKs from *Vitis vinifera* Chardonnay var. and Pinot Noir var. were acquired from the UniProt database [70]. Multiple sequence alignment was performed using the JalView v2.11.3.3 program with ClustalW default settings. Phylogenetic trees were constructed with the neighbor-joining (NJ) method [25,30]. 

### 4.5. Structure Prediction

Homology models of the lectin and kinase domains of all LecRLK proteins selected were obtained using the Phyre2.0 Protein Fold Recognition server (http://www.sbg.bio.ic.ac.uk/phyre2/html/page.cgi?id=index (accessed on 19 May 2024)). Confidence intervals of the generated models were over 95% in all cases [34]. 

### 4.6. Docking Analysis of Carbohydrate Interaction

The 3D structure of N-acetyl-galactosamine (GalNAc) was generated through the usage of CORINA Classic (https://demos.mn-am.com/corina_interactive.html (accessed on 9 July 2024) and adapted to an in vivo system with Antechamber v22.0 [44,45]. 

PDB files obtained from previous Phyre2 modeling were employed for the evaluation of potential binding sites with GalNAc using Autodock/Vina’s default settings. Ten docked ligand positions with the highest affinities were generated and manually contrasted with their corresponding original PDB structure. The ligand with the closest position to the original binding site and the highest affinity was chosen for each model [46]. 

## 5. Conclusions

Our findings emphasize the importance of pathogen recognition, specifically in the context of grapevines, as a critical step toward developing targeted treatments for diseases, such as downy mildew, caused by *Plasmopara viticola*. One of the key challenges is achieving these advancements while preserving the organoleptic properties of grape varieties, which are vital to the quality and market value of grape-derived products.

This research focused on lectin-type receptor-like kinases (LecRLKs) in *Vitis vinifera*, identifying 23 candidate lectin proteins associated with pathogen recognition in the grapevine varieties Chardonnay and Pinot Noir. These proteins were analyzed for their chromosome classification, evolutionary relationships, gene structure, and protein architecture. Despite these insights, challenges remain in defining the precise molecular mechanisms involved in pathogen recognition and signal transduction. Although docking experiments provided clues about potential carbohydrate-binding sites, the signaling pathways and molecular interactions require further investigation.

Looking ahead, future research should focus on unraveling these molecular mechanisms and exploring the functional roles of these proteins, particularly in relation to enhancing disease resistance in grapevines. This study also highlights the potential of using tandem duplication events in gene expansion to improve disease resistance. However, realizing these applications will require a deeper exploration of the signaling processes and interactions involved. Addressing these challenges will pave the way for innovative treatments that effectively combat grapevine pathogens while maintaining the essential qualities of grape varieties based on the development of host-specific defense elicitors.

## Figures and Tables

**Table 1:** Summary information of LecRLK genes from Chardonnay variety.

UniProt ID	Strand	Chromosome	Start	End	(bp)	ORF (aa)	Transcript ID Ensembl Plants *Vitis vinifera* (PN40024.v4)	Gene Identifier (Phytozome Genome ID *Vitis vinifera* v2.1)
A0A438F9N0	Forward	6	6,049,633	6,052,122	2490	675	Vitvi06g00512_P001	VIT_206s0004g05170 (PAC: 38062906)
A0A438GC44	Reverse	8	9,132,636	9,134,678	2043	490	Vitvi08g04118_t001	VIT_208s0058g00280 (PAC: 38023734)
A0A438GC81	Reverse	8	9,126,997	9,129,042	2046	682	Vitvi08g00740_t001	-
A0A438GC84	Reverse	8	9,161,497	9,163,515	2019	678	Vitvi08g00743_t001	-
A0A438GWD5	Reverse	8	9,165,420	9,167,591	2172	753	Vitvi08g00744_t001	VIT_208s0058g00290 (PAC: 38023825)
A0A438IQ18	Forward	8	13,330,945	13,334,344	3400	540	Vitvi08g01059_t001	VIT_208s0040g02210 (PAC: 38022968)
A0A438DE97	Reverse	8	15,007,752	15,009,794	2043	670	Vitvi08g01241_P001	VIT_208s0007g00810 (PAC: 38022999)
A0A438E3M7	Reverse	13	921,179	923,266	2088	695	Vitvi13g00096_P001	VIT_213s0067g01640 (PAC:38053159)
A0A438E3T4	Reverse	13	896,560	898,584	2025	628	Vitvi13g00095_P001	VIT_213s0067g01590 (PAC: 38053471)
A0A438J290	Reverse	13	885,704	887,737	2034	677	Vitvi13g04016_t001	VIT_213s0067g01640 (PAC: 38053159)
A0A438HKI7	Reverse	18	14,033,250	14,035,346	2097	671	Vitvi18g01267_t001	VIT_218s0166g00120 (PAC:38038848)
A0A438ER42	Forward	19	8,937,885	8,940,483	2599	679	Vitvi19g00690_P001	VIT_219s0015g00620 (PAC: 38059114)

**Table 2:** Summary information of LecRLK genes from Pinot Noir variety.

UniProt ID	Strand	Chromosome	Start	End	(bp)	ORF (aa)	Transcript ID Ensembl Plants *Vitis vinifera* (PN40024.v4)	Gene Identifier (Phytozome Genome ID *Vitis vinifera* v2.1)
F6H315	Reverse	4	4,770,650	4,773,320	1986	619	Vitvi04g01901_t001	VIT_204s0008g05320 (PAC: 38068899)
F6GUY7	Reverse	6	6,049,633	6,052,122	2490	675	Vitvi06g00512_t001	VIT_206s0004g05170 (PAC: 38062906)
F6HL80	Reverse	8	15,017,384	15,019,462	2079	692	Vitvi08g01243_t001	VIT_208s0007g00830 (PAC: 38025253)
F6HL81	Reverse	8	15,007,752	15,009,794	2043	680	Vitvi08g01241_t001	VIT_208s0007g00810 (PAC: 38022999)
F6HR07	Forward	8	13,330,945	13,334,344	3400	709	Vitvi08g01059_t001	VIT_208s0040g02210 (PAC: 38022968)
F6H1S2	Forward	12	15,204,429	15,206,366	1938	645	Vitvi12g01656_t001	VIT_212s0055g00500 (PAC: 38044712)
F6H5D8	Reverse	12	4,409,439	4,411,493	2055	1406	Vitvi12g00300_t001	VIT_212s0028g03580 (PAC: 38043165)
F6HNV3	Reverse	13	3,441,440	3,443,479	2040	679	Vitvi13g00332_t001	VIT_213s0019g02040 (PAC: 38053268)
F6HC85	Reverse	13	921,179	923,266	2088	673	Vitvi13g00096_t001	VIT_213s0067g01640 (PAC: 38053159)
F6HC87	Reverse	13	896,560	898,584	2025	674	Vitvi13g00095_t001	-
F6GSH6	Reverse	17	11,526,350	11,528,329	1980	659	Vitvi17g00905_t001	VIT_217s0000g09170 (PAC: 38030737)

**Table 3:** Carbohydrate specificity based on homology modeling. PDB homology code was obtained for each LecRLK sequence based on maximum confidence level and maximum sequence coverage. Asterisk corresponds to an incompletely modeled sequence. Gal: galactose; Gal-α-1, 3-Gal: galactose-alpha-1, 3-galactose; GluNAc: N-acetyl-glucosamine; GalNAc: N-acetyl-galactosamine; Man: mannose.

UniProt ID	PDB Homology Model Code	Carbohydrate Specificity	Reference
A0A438F9N0	3IPV	Gal	[35]
A0A438GC44	1DBN	Gal-α-1,3-Gal	[37]
A0A438GC81	1DBN	Gal-α-1,3-Gal	[37]
A0A438GC84	3IPV	Gal	[35]
A0A438GWD5	1AVB	GluNAc	[38]
A0A438IQ18	1F9K *	Man	[39]
A0A438DE97	3IPV	Gal	[35]
A0A438E3M7	2D3S	GalNAc	[40]
A0A438E3T4	3IPV	Gal	[35]
A0A438J290	2D3S	GalNAc	[40]
A0A438HKI7	2D3S	GalNAc	[40]
A0A438ER42	3IPV	Gal	[35]
F6H315	1DBN	Gal-α-1,3-Gal	[37]
F6GUY7	3IPV	Gal	[35]
F6HL80	1G8W	GluNAc	[41]
F6HL81	3IPV	Gal	[35]
F6HR07	2FMD	Man	[42]
F6H1S2	1DBN	Gal-α-1,3-Gal	[37]
F6H5D8	−2D3S/3IPV	GalNAc/Gal	[35,40]
F6HNV3	1LUI	GalNAc	[43]
F6HC85	2D3S	GalNAc	[40]
F6HC87	2D3S	GalNAc	[40]
F6GSH6	1G8W	GluNAc	[41]

**Table 4:** Affinity (Kcal/mol) calculated for each structure employing GalNAc as ligand. n.d.: non-determined.

UniProt ID	Affinity (kcal/mol)
A0A438E3M7	−4.8
A0A438HKI7	n.d.
A0A438J290	n.d.
F6HC85	−5.1
F6HC87	−5.6
F6HVN3	n.d.

## Data Availability

The original contributions presented in the study are included in the article/supplementary material; further inquiries can be directed to the corresponding author.

## Supplementary Materials

The following supporting information can be downloaded at: https://www.mdpi.com/article/10.3390/ijms25179553/s1.
